# Comparing progression biomarkers in clinical trials of early Alzheimer’s disease

**DOI:** 10.1002/acn3.51158

**Published:** 2020-08-11

**Authors:** Nicholas C. Cullen, Henrik Zetterberg, Philip S. Insel, Bob Olsson, Ulf Andreasson, the Alzheimer’s Disease Neuroimaging Initiative, Kaj Blennow, Oskar Hansson, Niklas Mattsson‐Carlgren

**Affiliations:** ^1^ Clinical Memory Research Unit Department of Clincal Sciences Malmö Faculty of Medicine Lund University Lund Sweden; ^2^ Department of Neurology Skåne University Hospital Sweden; ^3^ Wallenberg Center for Molecular Medicine Lund University Lund Sweden; ^4^ Institute of Neuroscience and Physiology Department of Psychiatry and Neurochemistry the Sahlgrenska Academy at the University of Gothenburg Mölndal Sweden; ^5^ Clinical Neurochemistry Laboratory Sahlgrenska University Hospital Mölndal Sweden; ^6^ Department of Neurodegenerative Disease UCL Institute of Neurology Queen Square London UK; ^7^ UK Dementia Research Institute at UCL London UK; ^8^ Department of Psychiatry University of California San Francisco CA US

## Abstract

**Objective:**

To investigate the statistical power of plasma, imaging, and cognition biomarkers as Alzheimer’s disease (AD) clinical trial outcome measures.

**Methods:**

Plasma neurofilament light, structural magnetic resonance imaging, and cognition were measured longitudinally in the Alzheimer’s Disease Neuroimaging Initiative (ADNI) in control (amyloid PET or CSF Aβ42 negative [Aβ‐] with Clinical Dementia Rating scale [CDR] = 0; n = 330), preclinical AD (Aβ + with CDR = 0; n = 218) and mild AD (Aβ + with CDR = 0.5‐1; n = 697) individuals. A statistical power analysis was performed across biomarkers and groups based on longitudinal mixed effects modeling and using several different clinical trial designs.

**Results:**

For a 30‐month trial of preclinical AD, both the temporal composite and hippocampal volumes were superior to plasma neurofilament light and cognition. For an 18‐month trial of mild AD, hippocampal volume was superior to all other biomarkers. Plasma neurofilament light became more effective with increased trial duration or sampling frequency. Imaging biomarkers were characterized by high slope and low within‐subject variability, while plasma neurofilament light and cognition were characterized by higher within‐subject variability.

**Interpretation:**

MRI measures had properties that made them preferable to cognition and pNFL as outcome measures in clinical trials of early AD, regardless of cognitive status. However, pNfL and cognition can still be effective depending on inclusion criteria, sampling frequency, and response to therapy. Future trials will help to understand how sensitive pNfL and MRI are to detect downstream effects on neurodegeneration of drugs targeting amyloid and tau pathology in AD.

## Introduction

Neurofilament light (NfL) is a cytoskeletal protein which is released from injured neurons in several neurodegenerative diseases, including in Alzheimer’s disease (AD).^1^, ^2^ NfL concentration measured in cerebrospinal fluid (CSF NfL) has served as a biomarker for axonal injury and neurodegeneration in research and to some degree clinical practice for decades, and recently a highly sensitive assay has been developed to reliably measure NfL in plasma (pNfL).^3^ Recent work has shown that pNfL levels increase over time in mild cognitive impairment and AD and are associated with brain atrophy in AD‐associated brain regions.^4^, ^5^

The development of an NfL assay in plasma follows a greater trend in the AD community focused on developing plasma biomarkers to measure β‐amyloid (Aβ)^6^, ^7^, ^8^ and tau pathologies.^9^, ^10^, ^11^, ^12^ Plasma biomarkers have the potential to transform the tracking of biological effects of drugs in clinical trials by significantly reducing common barriers for sample collection such as high measurement cost, time‐consuming procedures (*e.g*., MRI), or aversion to lumbar puncture.^3^

Still, the statistical power of pNfL as a potential marker of treatment effect in AD clinical trials has not been thoroughly assessed, particularly in relation to magnetic resonance imaging (MRI) and cognition. Power is commonly used as the basis by which the minimum number of clinical trial participants is determined and has previously been used in trials of AD.^13^ In the present study, we assess statistical power from using longitudinal pNfL, structural MRI, and cognition as outcome measures in both preclinical and mild AD – two groups which hold strong relevance for future clinical trials.

## Methods

### Participants

The dataset used for all analyses was obtained from the database of the Alzheimer’s Disease Neuroimaging Initative (ADNI),^14^ which was launched in 2003 as a public‐private partnernship. Participants in the ADNI study have been recruited from more than 50 locations across the United States and Canada. Regional ethics committees of all institutions approved the ADNI study and all study participants gave written informed consent.

Inclusion and exclusion criteria for ADNI have been described in detail previously.^14^ Briefly, all ADNI participants were between the ages of 55 and 90 years, had completed at least six years of education, were fluent in Spanish or English, and had no significant neurologic disease other than AD. All subjects with a CDR score of 0 (no significant cognitive impairment), 0.5 (very mild dementia), or 1 (mild dementia) who had 18F‐florbetapir (amyloid) PET or CSF Aβ42 data available were eligible. Amyloid status was defined primarily using PET (described below), while amyloid status for those without PET data was determined using CSF Aβ42 (using a fully automated Elecsys immunoassay, Roche Diagnostics) with a cutoff of 880 pg/mL as defined previously ^4^. Participants in this study were also required to have at least two follow‐up measures of MRI, pNfL, and cognition.

Data for this study were downloaded on 15 December 2019 using the following files: “ADNIMERGE.csv”, “ADNI_BLENNOWPLASMANFLLONG_10_03_18.csv”, “UCSFFSX51FINAL_11_08_19.csv”, “CDR.csv”, “UCBERKELEYAV45_08_27_19.csv”, and “UPENNBIOMK9_04_19_17.csv”. The study data and samples were collected from 7 September 2005 through 4 March 2019.

### Image acquisition and processing

Structural MRI brain scans were acquired using a 3T scanner with a standardized protocol that included collecting T1‐weighted images using a sagittal, volumetric, magnetization‐prepared rapid acquisition with gradient echo sequence. Measurements of regional volume and thickness according to the 2010 Desikan‐Killany atlas were obtained using an automated longitudinal pipeline in FreeSurfer (v5.1).^15^ MRI brain scans collected at the 1.5T strength in the earliest phase of ADNI were not included in this analysis.

From structural brain images, a temporal composite previously identified as closely relating to AD progression and consisting of the average area‐normalized bilateral cortical thickness in entorhinal, fusiform, inferior temporal, and middle temporal regions was extracted for use in power analysis comparisons.^16^ White matter hyperintensities were quantified using an automated pipeline with fluid‐attenuated inversion recovery images as input.^17^

18F‐Florbetapir PET brain scans for Aβ deposition in the brain were processed and analyzed at the University of California at Berkeley according to a previously described protocol^18^ and SUVR values were derived from a cortical ROI consisting of frontal, anterior/posterior cingulate, lateral parietal, lateral temporal brain regions and with a composite reference region made up of whole cerebellum, brainstem/pons, and eroded subcortical white matter optimized for longitudinal analysis. Because many study participants lacked an 18F‐Florbetapir PET scan at their initial baseline study visit, 18F‐florbetapir PET status for each individual at baseline was instead estimated using all available longitudinal amyloid PET scans. To achieve this, a linear regression model was fit on all available longitudinal SUVR values for each individual separately and the resulting model intercept (representing estimated 18F‐florbetapir PET SUVR at baseline) was extracted and compared to a predefined cutoff for 18F‐florbetapir PET positivity (SUVR intercept> 0.79 indicating amyloid positivity)^19^. This method made it possible to anchor amyloid PET status to the common study baseline visit when biomarker collection began.

### Plasma NfL quantification

Concentration of pNfL was measured at the Clinical Neurochemistry Laboratory, University of Gothenburg, Sweden, using an in‐house ultrasensitive enzyme‐linked immunosorbent assay on the Single molecule array platform (Quanterix Corp, Lexington, MA, USA), as previously described in detail.^20^ The assay had lower and upper limits of quantifications of 6.7 ng/L and 1620 ng/L, respectively. The intra‐ and inter‐assay coefficients of variation were 6.2% and 9.0%, respectively, for the low‐concentration quality control (QC) sample (11 ng/L), and 4.9% and 7.2%, respectively, for the high‐concentration QC sample (173 ng/L). The measurements were performed in January‐April 2018 by a board‐certified laboratory technician using a single batch of reagents.

### Cognitive measures

The cognitive measures included in the current analysis were the Clinical Dementia Rating – Sum of Boxes (CDRSB), which reflects clinically relevant symptoms throughout AD progression, and the Preclinical Alzheimer Cognitive Composite (PACC),^21^ which is currently used in clinical trials aimed at preclinical AD. PACC is an equally weighted sum of four components – the Mini‐Mental State Examination (MMSE), Logical Memory Delayed Recall (dMemory), Trail‐Making Test B (Trials B), and the Delayed Word Recall for the Alzheimer’s Assessment Scale ‐ Cognitive Subscale (dADASc) – chosen from prior literature because they demonstrate a close association with early alterations in the disease process, including episodic memory, executive function, orientation, and language.

### Study groups

Three separate subgroups were analyzed here from the overall cohort based on amyloid status as determined by 18F‐florbetapir PET (or CSF Aβ42 if PET was not available) and cognition as measured by the CDR Global cognitive scale. The first group (“controls”) comprised individuals with no cognitive impairment (CDR = 0) who were amyloid‐negative (Aβ‐). The second group (“preclinical AD”) comprised individuals who have no cognitive impairment (CDR = 0) but were amyloid‐positive (Aβ+). The final group (“mild AD”) comprised individuals with cognitive impairment (CDR = 0.5 or 1, representing “very mild” or “mild” dementia) who were also amyloid‐positive (Aβ+). A sensitivity analysis was performed with a requirement that mild AD individuals also showed altered levels of CSF P‐tau181 (measured by an Elecsys assay [Roche Diagnostics GmbH, Penzberg, Germany]; P‐tau was defined as positive when CSF P‐tau181> 27 using a previously published cutoff^22^ in addition to being Aβ‐positive. These groups are relevant for current AD trials which have a heightened focus on early disease stages and acknowledge an increased willingness of regulatory agencies to recognize early staging of AD through the coupling of cognition and biomarkers of Aβ pathology.^16^

### Statistical analysis

To evaluate the effectiveness of the relevant biomarkers in a clinical trial scenario, a power analysis was performed to determine the sample size needed to achieve 80% power when assuming a treatment effect of 30% reduction in expected longitudinal progression. Clinical trial duration was assumed to be 30 months for preclinical AD and 18 months for mild AD, with sampling frequency of one month for pNfL and three months for MRI and cognitive measures. The selection of a standard clinical trial duration is based on a review of recent clinical trials demonstrating an average treatment exposure duration of 73 weeks for Phase III trials of mild AD and 112 weeks for preclinical AD.^21^ The choice of plasma sampling frequency reflects the fact that recent trials of antiamyloid drugs have been characterized by an infusion approximately every month.^21^ A sensitivity analysis was performed with a plasma sampling frequency of three months and an MRI and cognitive sampling frequency of six months.

Difference in timing of measurements across data modalities was handled by adjusting for time from baseline in all models. Due to differences in number of follow‐up visits across modalities as a potential cofounder, a sensitivity analysis was performed in which only overlapping longitudinal data were included.

Power was calculated based on a previously established formula adapted for linear mixed effects (LME) models ^13^, ^23^, and which considers clinical trial structure, fixed (group‐averaged) effects, and random (individual‐specific) effects:

$$n\;per\;arm=\frac{2\left(\sigma_{w}^{2}+\frac{\sigma_{b}^{2}}{\Sigma {\left(t_{j}‐\bar{t}\right)}^{2}}\right){\left(z_{1‐\frac{\alpha }{2}}+z_{1‐\beta }\right)}^{2}}{\Delta^{2}}$$

where terms with z‐scores represent the standard normal distribution values corresponding to the desired significance level (*e.g*., *α* = 0.05) and power (*e.g.,* *β* = 0.8), the $\Sigma {\left(t_{j}‐\bar{t}\right)}^{2}$ term takes into account trial duration and biomarker sampling frequency, $\sigma_{w}^{2}$ is the within‐subject variance (*i.e*., the variability of observations around each individual’s estimated slope) of the fitted LME model, $\sigma_{b}^{2}$ is the between‐subject variance (*i.e*., variability of all individual’s estimated slopes) of the fitted LME model, and Δ is either the group‐averaged slope of the fitted LME model in the treatment group (when assuming a treatment mechanism in which biomarker change over time can be reduced to zero) or the difference in slope of the fitted LME model between the treatment group and the control group (when assuming a treatment mechanism in which biomarker change over time can be at most reduced to levels seen during normal aging). Significant differences in the sample size needed to achieve adequate trial power using different outcome measures were assessed using a standard bootstrapping procedure (n = 250 iterations).

An additional analysis was performed in which both clinical trial duration and biomarker sampling frequency were allowed to vary within a reasonable range in order to understand the effect of trial conditions on power calculations. Differences in sample sizes between pNfL and each other biomarker for each possible combination of trial duration and sampling were assessed using bootstrapping (n = 250 iterations).

All statistical analyses were carried out using the R programming language (v3.5.1) with LME modeling performed using the *nlme* (v3.1) package and the power analysis procedure performed using the *longpower* (v1.0) package. All tests were two‐sided with a significant level set to *P* < 0.05.

## Results

### Cohort and subgroup characteristics

The groups analyzed (Table 1) included a control group (n_total_ = 330, obs_MRI_ = 631, obs_pNfL_ = 750, obs_COG_ = 1,794), a preclinical AD group (n_total_ = 218, obs_MRI_ = 343, obs_pNfL_ = 430, obs_COG_ = 1,117), and a mild AD group (n_total_ = 697, n_tau+_ = 388, obs_MRI_ = 1,510, obs_pNfL_ = 1,457, obs_COG_ = 3,559). The groups did not significantly differ on sex, education, or age. Individuals in the preclinical AD group had similar cognitive status as the control group but higher rates of abnormal AD‐associated CSF biomarker levels.

**Table 1:** Cohort demographics and sample counts

Characteristic	Controls [Aβ‐, CDR = 0]	Preclinical AD [Aβ+, CDR = 0]	Mild AD [Aβ+, CDR = 0.5,1]
Total N	330	218	697
Age at baseline, mean (SD)	72.7 (6.1)	73.3 (6.1)	73.4 (7.2)
Male sex, No. (%)	165 (50.0%)	83 (38.1%)	394 (56.5%)
Education level, mean (SD)	16.2 (2.5)	16.4 (2.5)	15.7 (2.8)
CSF biomarkers at baseline, mean (SD)			
Aβ42	1452.9 (311.9)	878.2 (415.9)	676.9 (266.8)
t‐tau	211.1 (69.5)	270.5 (105.8)	342.9 (141.6)
p‐tau	18.7 (6.2)	26.1 (11.1)	34.5 (15.4)
Cognitive score at baseline, mean (SD)			
MMSE	29.2 (1.1)	29.0 (1.2)	26.0 (2.7)
CDRSB	0.02 (0.11)	0.04 (0.12)	2.5 (1.6)
PACC	0.05 (2.6)	−0.35 (2.8)	−9.8 (6.2)
pNfL/ MRI/ Cognition, No. samples			
Baseline	171/ 120/ 330	114/ 74/ 218	391/ 306/ 697
Follow‐up, months			
6	0/ 93/ 247	0/ 53/ 166	2/ 290/ 615
12	133/ 105/ 221	66/ 54/ 146	335/ 285/ 600
24	141/ 99/ 234	84/ 55/ 149	229/ 184/ 444
36	13/ 4/ 96	8/ 2/ 62	167/ 39/ 293
48	76/ 49/ 143	37/ 21/ 78	124/ 68/ 202
60	43/ 7/ 65	26/ 2/ 43	50/ 11/ 126

Continuous data are reported as mean (standard deviation). CSF biomarkers (measured in pg/ml), imaging measures and cognitive summaries were calculated from original baseline study visit. Visit codes are in relation to overall study participation. In the Mild AD group, 576 of 697 participants had CDR = 0.5, and 121 of 697 participants had CDR = 1.0.

**Figure 1:**
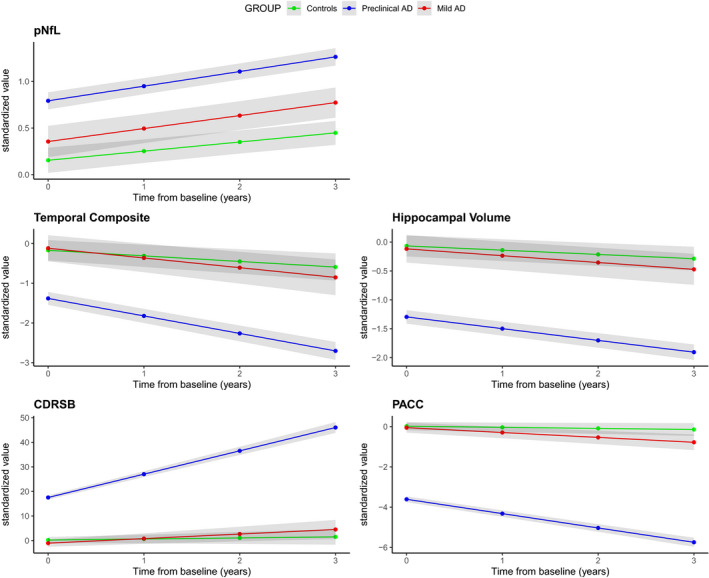
Longitudinal trajectories of pNfL across groups. This figure shows the predicted longitudinal trajectories of pNfL, AD‐signature cortical thickness temporal composite, bilateral hippocampal volume, CDRSB, and PACC

In the preclinical AD group, pNfL levels did not increase significantly faster over time compared with controls (Δβ = 0.04 sd./ year, *P* = 0.10) and were not significantly higher at baseline compared with controls (Δβ = 0.21 sd., *P* = 0.07). All other biomarkers (temporal composite and hippocampal volume for MRI; CDRSB, and PACC for cognition) had significantly greater rates of change in preclinical AD compared with controls (Figure 1). In the mild AD group, pNfL levels did increase at a significantly faster rate compared with controls (Δβ = 0.06 sd./ year, *P* = 0.002) and were significantly higher at baseline compared with controls (Δβ = 0.64 sd., *P* < 0.001). All other biomarkers also had significantly greater rates of change in mild AD compared with controls (Figure 1).

### Power analysis in a standard clinical trial scenario

**Figure 2:**
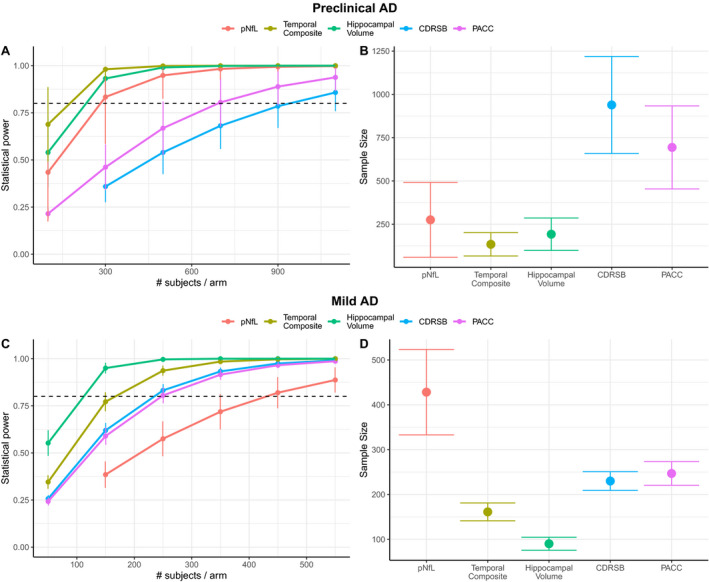
Standard Power analysis. This figure shows the power analysis comparison between pNfL, imaging, and cognition biomarkers. Subpanel A, respectively C, shows for preclinical AD, respectively mild AD, the statistical power to observe a 30% decrease in progression as a function of sample size while subpanel B, respective D, shows for preclinical AD, respectively mild AD, the sample size needed to achieve 80% power to observe a 30% decrease in progression. Error bars were calculated using 100 bootstrapped samples.

In a 30‐month clinical trial of preclinical AD (Figure 2A‐B), using pNfL as a progression marker would require 289 subjects (CI [76, 496]) to achieve 80% power to detect 30% treatment effect, while temporal composite would require 125 subjects (CI [78, 172]; *P* < 0.0001 signficantly less than pNfL), hippocampal volume would require 184 subjects (CI [91, 278]; *P* < 0.0001 signficantly less than pNfL), CDRSB would require 939 subjects (CI [634, 1245]; *P* < 0.0001 signficantly more than pNfL), and PACC would require 669 subjects (CI [430, 909]; *P* < 0.0001 signficantly more than pNfL).

In an 18‐month clinical trial of mild AD (Figure 2C‐D), using pNfL as a progression marker would require 432 subjects (CI [334, 529]), while temporal composite would require 161 subjects (CI [140, 182]; *P* < 0.0001 compared with pNfL), hippocampal volume would require 90 subjects (CI [76, 104]; *P* < 0.0001 compared with pNfL), CDRSB would require 230 subjects (CI [211, 249]; *P* < 0.0001 signficantly less than pNfL), and PACC would require 249 subjects (CI [220, 277]; *P* < 0.0001 signficantly less than pNfL). In a sensitivity analysis where mild AD individuals were additionally required to also be P‐tau+ (n = 388), the results were qualitatively similar but the number of required participants decrease throughout. Here, using pNfL as a progression marker would require 406 subjects (CI [295, 516]), while temporal composite would require 109 subjects (CI [90, 127]), hippocampal volume would require 60 subjects (CI [49, 70]), CDRSB would require 175 subjects (CI [155, 195]), and PACC would require 166 subjects (CI [146,185]).

To determine whether number of longitudinal follow‐up measures available across biomarkers were confounding power analysis results, we performed the same analysis as above using only time points for each participant in which all biomarkers were available. Because cognitive measures had the most timepoints, this served to mostly reduce cognitive follow‐ups and resulted in a reduced effectiveness of cognitive measures. In preclinical AD, there was no significant difference between pNfL and cognitive measures, as CDRSB required 1104 participants (CI [227, 1981]) and the PACC model did not converge due to having such high variability, while pNfL required 651 participants (CI [0, 1665]), temporal composite required 182 participants (CI [75, 289]) and hippocampal volume required 177 participants (CI [76, 278]). In mild AD, there was also no significant difference between pNfL and cognitive measures, as CDRSB now required 314 participants (CI [246, 381]) and PACC required 385 participants (CI [288, 481]), while pNfL required 429 participants (CI [265, 592]), temporal composite required 174 participants (CI [137, 211]) and hippocampal volume required 98 participants (CI [83, 114]).

### Effect of assumed treatment mechanism on power

The primary power analysis above assumed a hypothetical treatment mechanism in which a therapy could potentially reduce biomarker change over time to zero and thus biomarker change during normal aging was not considered. An additional analysis was performed here in which a treatment mechanism could affect only disease‐specific biomarker progression, *i.e*., changes above and beyond the progression found in healthy individuals during normal aging. Thus, the reduction in slope of a given biomarker was calculated as 30% of the estimated slope in the treatment group (preclinical or mild AD) *minus* the estimated slope in the control group.

**Figure 3:**
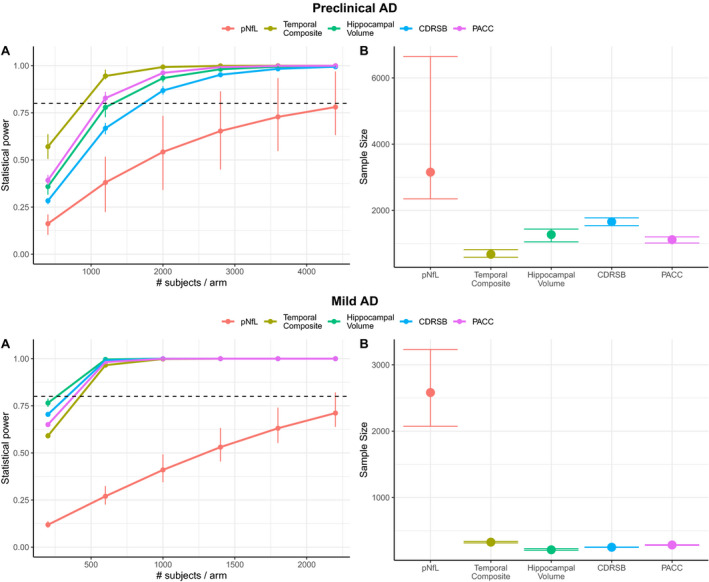
Alternative Mechanism Power analysis. This figure shows the power analysis comparison between pNfL, imaging, and cognition biomarkers, under the assumption of an alternative treatment mechanism targets only disease‐specific biomarker change above and beyond that seen in the control group. Subpanel A, respectively C, shows for preclinical AD, respectively mild AD, the statistical power to observe a 30% decrease in progression as a function of sample size while subpanel B, respective D, shows for preclinical AD, respectively mild AD, the sample size needed to achieve 80% power to observe a 30% decrease in progression. Error bars were calculated using 100 bootstrapped samples.

In such a sensitivity analysis for a 30‐month trial of preclinical AD (Figure 3A‐B) using pNfL as a progression marker would require 2909 subjects (CI [2306, 4219]), while temporal composite would require 711 subjects (CI [556, 820]; *P* < 0.0001 compared with pNfL), hippocampal volume would require 1303 subjects (CI [1140, 1497]; *P* < 0.0001 compared with pNfL), CDRSB would require 1745 subjects (CI [1576, 1938]; *P* < 0.0001 signficantly less than pNfL), and PACC would require 1110 subjects (CI [1024, 1184]; *P* < 0.0001 signficantly less than pNfL).

For an 18‐month clinical trial of mild AD (Figure 3C‐D), using pNfL as a progression marker would require 432 subjects (CI [334, 529]), while temporal composite would require 161 subjects (CI [140, 182]; *P* < 0.0001 compared with pNfL), hippocampal volume would require 90 subjects (CI [76, 104]; *P* < 0.0001 compared with pNfL), CDRSB would require 230 subjects (CI [211, 249]; *P* < 0.0001 signficantly less than pNfL), and PACC would require 249 subjects (CI [220, 277]; *P* < 0.0001 signficantly less than pNfL).

### Effect of trial duration and sampling frequency on power

**Figure 4:**
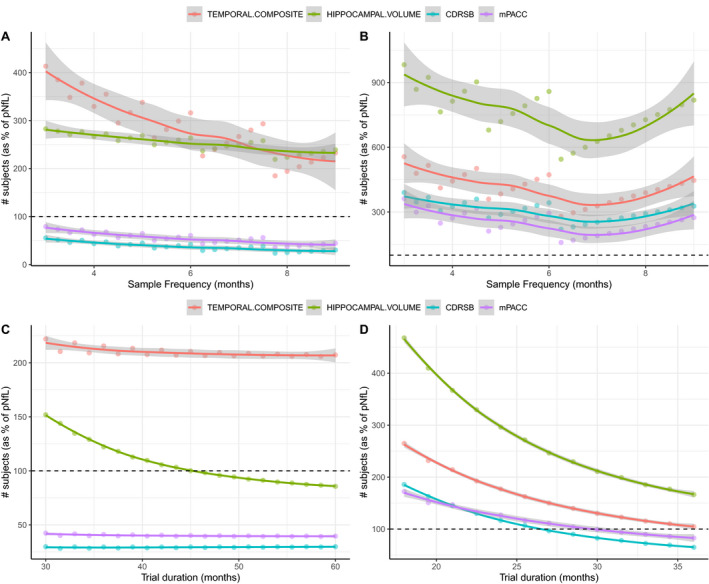
Effect of clinical trial structure on power calculations. This figure shows the results after varying the clinical trial duration and frequency of follow‐up on power calculations in preclinical and mild AD. Subpanel A and B show – for preclinical AD and mild AD, respectively – the number of subjects (relative to pNfL) required to achieve 80% power to detect 30% reduction in progression as the sample frequency of pNfL was fixed at once per month but was varied for all other biomarkers from three to nine. Subpanels C and D show – for preclinical AD and mild AD, respectively – the number of trial subjects required (relative to pNfL) as trial duration is varied. Values greater than 1.0 indicate that the biomarker requires *less* subjects than pNfL while values less than 1.0 indicate that the biomarker requires *more* subjects than pNfL.

A further experiment was carried out to test the effect of biomarker sampling frequency and clinical trial length on power. When the sampling frequency of pNfL was fixed to every one month, while sampling frequency of MRI and cognition systematically varied between three and nine months, pNfL was always superior to cognition and was noninferior (neither significantly better nor worse) than MRI‐based measures when MRI scans were taken at most every nine months in preclinical AD (Figure 4A). In mild AD, pNfL was noninferior to cognition given that cognition was evaluated at most every seven months (Figure 4B). The results were similar when performing the same experiment but with the sampling frequency of pNfL fixed to every three months.

Moreover, when clinical trial duration was systematically varied between 30 and 60 months in preclinical AD while keeping sampling frequency fixed (Figure 4C), pNfL was consistently better than both cognition measures and became preferable to hippocampal volume when trial duration was greater than 45 months. For mild AD, pNfL became preferable to cognition for trial durations greater than 28 months and preferable to the temporal composite for trials greater than 36 months (Figure 4D).

### Characterizing factors related to longitudinal data

Besides the setup of the clinical trial (duration and sampling frequency), power results are also greatly influenced by inherent characteristics of the longitudinal data itself – namely, average group slope, within‐subject variability (how much an individual’s data points vary around that individual’s estimated slope), and between‐subject variability (how much all individual’s estimated slope vary around the average group slope).

**Figure 5:**
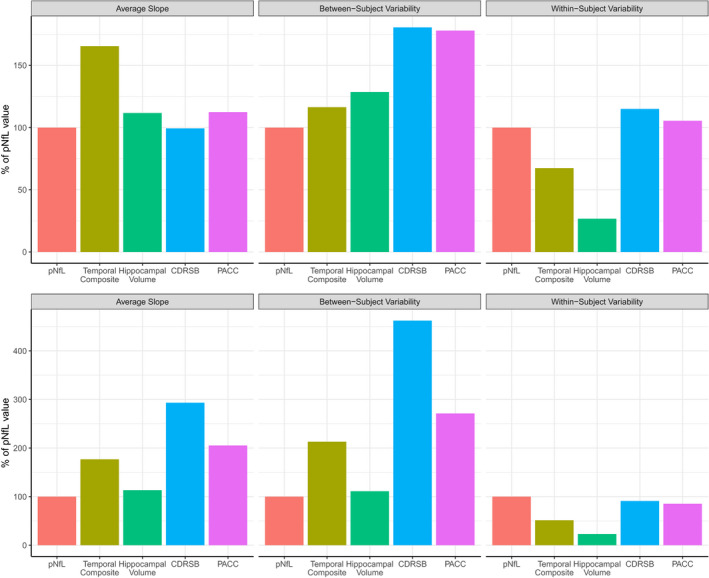
Longitudinal data characteristics affecting power. This figure shows the average slope, between‐subject variability, and within‐subject variability estimated across all biomarkers for both mild AD (top panel) and preclinical AD (bottom panel). The characteristics shown here include average group slope, within‐subject variability (how much an individual’s data points vary around that individual’s estimated slope), and between‐subject variability (how much all individual’s estimated slope vary around the average group slope).

Analyzing these characteristics across biomarkers in preclinical AD showed that pNfL had a higher within‐subject variability while also having a lower change over time (slope) than MRI measures (Figure 5, upper panel). This phenomenon was also seen in the mild AD group (Figure 5, bottom panel). Additionally, the within‐subject variability levels of pNfL were comparable to cognition measures for both mild and preclinical AD, while the average slope was lower for pNfL in the mild AD group. Moreover, pNfL also had the lower between‐subject variability across biomarkers for both disease groups.

## Discussion

Through a comprehensive power analysis, we have demonstrated that pNfL, cognition or imaging cannot be considered a “silver bullet” when it comes to powering clinical trials of early AD. In simulations of trials of mild AD, pNfL would likely require more participants to achieve equivalent statistical power to MRI and cognition. However, a sensitivity analysis using only overlapping data showed that the estimated benefit in power of cognition over pNfL may be caused by cognitive measures having reduced variability due to more follow‐up data. We also showed that trial structure is an important factor to consider, as biomarkers with high within‐subject variability but low between‐subject variability (*e.g*., pNfL) perform better relative to other biomarkers as the duration of a clinical trial increases and when pNfL is sampled more often relative to MRI and cognitive measures. This is an important benefit for pNfL (and other blood‐based biomarkers), since it may be more feasible to increase the frequency of blood sampling in a clinical trial, compared to increasing frequencies of MRI scans; our results show that sampling pNfL every one to three months will be most effective.

The largest benefits of MRI markers were the low within‐subject variability and high change over time, especially relative to healthy controls in the alternative treatment mechanism. The low within‐subject variability shows that individual’s data points largely follow a known trajectory over time once a few data points are collected. Subsequently, it means that MRI is not required to be sampled as frequently as for other biomarkers.

Conversely, the largest hinderances for pNfL and cognition as progression markers were levels of within‐subject variability observed in the disease groups. For pNfL, there was also a high degree of change seen in healthy individuals. High within‐subject variability is less likely to be attributed to long‐term, AD‐related heterogeneity and is instead more closely related to short‐term, biological rhythms and technical variability. Adjustment for white matter damage did not account for this variability in pNfL.

Moreover, we have previously quantified the interassay coefficient of variation for pNfL in the current cohort to be 9%, and in our current analysis we found that this technical variability constituted around 15% of the total within‐subject variability found in the longitudinal pNfL data. Reduction of preanalytical variability could therefore be beneficial. Importantly, samples of pNfL were completely randomized during assay measurement in ADNI, meaning that samples from the same individuals ended up on different plates and within‐subject variability was likely inflated. As in other studies of pNfL, in a real clinical trial samples from the same individual should be analyzed side‐by‐side on the same plate in order to minimize within‐subject variability ^24^, ^25^.

It is essential to note that a power analysis is only as good as the assumptions which underlie it. Namely, our assumption that a treatment would result in a 30% reduction in the expected progression of a biomarker, and that each biomarker would be affected by 30%, can be called into question. In fact, results from recent clinical trials for AD may cast doubt on whether a 30% reduction is truly possible for MRI‐based biomarkers, with some trials actually reporting *increased* rates of expected brain volume loss in treatment groups, despite some evidence of target engagement on Aβ.^26^, ^27^ Clearing Aβ from the brain may therefore cause a (possibly temporary) reduction in brain volume – it is unknown if this reflects actual loss of tissue, or changes in volume due to other processes – in which case structural brain imaging is unlikely to be useful in monitoring treatment effects on neurodegeneration from an anti‐Aβ treatment.

These MRI‐findings stand in stark contrast with recent trials of multiple sclerosis, in which fluid levels of NfL were in fact shown to respond to treatment and to be associated with improved clinical outcomes.^28^, ^29^ Similarly, NfL measured in CSF shows a very distinct treatment response with antisense oligonucleotide treatment in children with spinal muscular atrophy.^30^ Together, those successful results motivate further considerations of NfL as an outcome marker also in AD clinical trials, to evaluate how sensitive this biomarker is to identify downstream effects on neurodegeneration by drug candidates targeting amyloid and tau pathology.

Although NfL levels are greatly increased in other neurodegenerative disorders compared with AD,^31^ it may still be the case that a fluid marker like pNfL can simply pick up treatment effects much more dynamically than imaging biomarkers could. Interestingly, this effect has actually been reported to occur in recent anti‐tau trials in AD.^32^ Additionally, since cognition is the primary outcome marker for all current AD clinical trials, it is unlikely to be replaced before at least one treatment is shown to improve cognitive outcomes.

Another important assumption underlying such a power analysis is that the study population accurately represents clinical trial participants. Our selection of participants at the early stages of disease may favor MRI measures, as does the fact that highly educated individuals (as found in the ADNI cohort) undergo less cognitive change on the group level than what would be seen in a more heterogeneous population. Still, the participants used in our current analysis are likely quite similar to the type of individuals who are part of current clinical trials of AD – i.e., individuals with evidence of Aβ accumulation but without significant cognitive decline. An analysis into mild AD individuals who showed both Aβ and tau positivity showed the same qualitative results, although cognition became more closer to MRI measures in effectiveness (and required sample sizes were generally smaller, due to more pronounced longitudinal changes in this more highly selected group). In general, our results may differ if we were to look in the context of progression markers in the general population after a disease‐altering therapy is available. We speculate that outside of a clinical trial context, both cognitive measures and pNfL might be more important since the likely patient comes from a more heterogenous population.

In all, more data are needed to understand the extent to which pNfL and MRI respond to disease‐modifying drug candidates and the source of variability of pNfL over time within the same individual. Understanding how the benefit of additional blood or MRI measurements differs depending on when in a clinical trial they are taken (e.g., sampling more often at the beginning or towards the end of a trial) is also an important direction for future work. However, only once a treatment is found which reduces expected change in cognition can pNfL and MRI be fully considered as primary markers. Novel plasma biomarkers can also be considered for future clinical trial design, including for beta‐amyloid^7^, ^8^ and tau.^10^, ^11^ However, although the AD‐specific changes will certainly be important progression markers in AD clinical trials, biomarkers such as pNfL and MRI are still needed to track the ultimate downsteam effect of potential therapies, namely the reduction or halting of neurodegeneration.22.

## Consent for publication

Not applicable.

## Ethics approval and consent to participate

Consent was received from all individuals prior to participation in the ADNI study. Ethics approval was received by institutional review boards at each participating site.

## Data Availability Satatement

All data is available online on the official ADNI website. Preprocessing and analysis code is available upon request.

## Conflict of interest

HZ has served at scientific advisory boards for Denali, Roche Diagnostics, Wave, Samumed and CogRx, has given lectures in symposia sponsored by Biogen, Fujirebio and Alzecure, and is a cofounder of Brain Biomarker Solutions in Gothenburg AB, a GU Ventures‐based platform company at the University of Gothenburg. OH has acquired research support (for the institution) from Roche, GE Healthcare, Biogen, AVID Radiopharmaceuticals and Euroimmun. In the past 2 years, he has received consultancy/speaker fees (paid to the institution) from Biogen and Roche. KB has served as a consultant or at advisory boards for Abcam, Axon, Biogen, Lilly, MagQu, Novartis and Roche Diagnostics, and is a cofounder of Brain Biomarker Solutions in Gothenburg AB, a GU Ventures‐based platform company at the University of Gothenburg.

## Funding Information

Work at the authors’ research center was supported by the Wallenberg Center for Molecular Medicine, the Knut and Alice Wallenberg foundation, The Medical Faculty at Lund University, Region Skåne, Swedish Research Council, the Marianne and Marcus Wallenberg foundation, the Strategic Research Area MultiPark (Multidisciplinary Research in Parkinson’s disease) at Lund University, the Swedish Alzheimer Foundation, the Swedish Brain Foundation, The Parkinson foundation of Sweden, the Swedish Medical Association, the Konung Gustaf V:s och Drottning Victorias Frimurarestiftelse, The Bundy Academy, The Parkinson Research Foundation, the Skåne University Hospital Foundation, and the Swedish federal government under the ALF agreement. The Clinical Neurochemistry Laboratory, University of Gothenburg is supported by the Swedish Research Council (#2017‐00915; #2018‐02532), the Alzheimer Drug Discovery Foundation (ADDF), USA (#RDAPB‐201809‐2016615), the European Research Council (#681712), the Swedish Alzheimer Foundation (#AF‐742881), Hjärnfonden, Sweden (#FO2017‐0243), the Swedish state under the agreement between the Swedish government and the County Councils, the ALF agreement (#ALFGBG‐715986; #ALFGBG‐720931). Data collection and sharing was funded by the Alzheimer's Disease Neuroimaging Initiative (ADNI) (National Institutes of Health Grant U01 AG024904) and DOD ADNI (Department of Defense award number W81XWH‐12‐2‐0012). ADNI is funded by the National Institute on Aging, the National Institute of Biomedical Imaging and Bioengineering, and through generous contributions from the following: AbbVie, Alzheimer’s Association; Alzheimer’s Drug Discovery Foundation; Araclon Biotech; BioClinica, Inc.; Biogen; Bristol‐Myers Squibb Company; CereSpir, Inc.; Cogstate; Eisai Inc.; Elan Pharmaceuticals, Inc.; Eli Lilly and Company; EuroImmun; F. Hoffmann‐La Roche Ltd and its affiliated company Genentech, Inc.; Fujirebio; GE Healthcare; IXICO Ltd.; Janssen Alzheimer Immunotherapy Research & Development, LLC.; Johnson & Johnson Pharmaceutical Research & Development LLC.; Lumosity; Lundbeck; Merck & Co., Inc.; Meso Scale Diagnostics, LLC.; NeuroRx Research; Neurotrack Technologies; Novartis Pharmaceuticals Corporation; Pfizer Inc.; Piramal Imaging; Servier; Takeda Pharmaceutical Company; and Transition Therapeutics. The Canadian Institutes of Health Research is providing funds to support ADNI clinical sites in Canada. Private sector contributions are facilitated by the Foundation for the National Institutes of Health (www.fnih.org). The grantee organization is the Northern California Institute for Research and Education, and the study is coordinated by the Alzheimer’s Therapeutic Research Institute at the University of Southern California. ADNI data are disseminated by the Laboratory for Neuro Imaging at the University of Southern California.

## Authors’ contributions

Nicholas C. Cullen performed the statistical analysis, study design, and drafting of manuscript. Henrik Zetterberg contributed to data collection, study design, and revision of manuscript. Philip S. Insel contributed to the statistical analysis and revision of manuscript. Bob Olsson contributed to the study design and revision of manuscript. Ulf Andreasson contributed to data collection, study design and revision of manuscript. Kaj Blennow contributed to data collection, study design, and revision of manuscript. Oskar Hansson contributed to the study design and revision of manuscript. Niklas Mattsson‐Carlgren contributed to thw statistical analysis, study design, and drafting of manuscript.
